# Expectant Mothers Maximizing Opportunities: Maternal Characteristics Moderate Multifactorial Prenatal Stress in the Prediction of Birth Weight in a Sample of Children Adopted at Birth

**DOI:** 10.1371/journal.pone.0141881

**Published:** 2015-11-06

**Authors:** Line Brotnow, David Reiss, Carla S. Stover, Jody Ganiban, Leslie D. Leve, Jenae M. Neiderhiser, Daniel S. Shaw, Hanna E. Stevens

**Affiliations:** 1 Dept of Psychiatry, University of Iowa College of Medicine, Iowa City, IA, United States of America; 2 Yale Child Study Center, Yale School of Medicine, New Haven, CT, United States of America; 3 Dept. of Mental Health Law & Policy, University of South Florida, Tampa, FL, United States of America; 4 Dept. of Psychology, George Washington University, Washington DC, United States of America; 5 Dept. of Counseling Psychology & Human Services, University of Oregon, Eugene, OR, United States of America; 6 Dept. of Psychology, Penn State University, University Park, PA, United States of America; 7 Dept. of Psychology, University of Pittsburgh, Pittsburgh, PA, United States of America; Technion - Israel Institute of Technology, ISRAEL

## Abstract

**Background:**

Mothers’ stress in pregnancy is considered an environmental risk factor in child development. Multiple stressors may combine to increase risk, and maternal personal characteristics may offset the effects of stress. This study aimed to test the effect of 1) multifactorial prenatal stress, integrating objective “stressors” and subjective “distress” and 2) the moderating effects of maternal characteristics (perceived social support, self-esteem and specific personality traits) on infant birthweight.

**Method:**

Hierarchical regression modeling was used to examine cross-sectional data on 403 birth mothers and their newborns from an adoption study.

**Results:**

Distress during pregnancy showed a statistically significant association with birthweight (R^2^ = 0.032, *F*_(2, 398)_ = 6.782, *p* = .001). The hierarchical regression model revealed an almost two-fold increase in variance of birthweight predicted by stressors as compared with distress measures (R^2^ *Δ* = 0.049, *F*_(4, 394)_ = 5.339, *p* < .001). Further, maternal characteristics moderated this association (R^2^ *Δ* = 0.031, *F*_(4, 389)_ = 3.413, *p* = .009). Specifically, the expected benefit to birthweight as a function of higher SES was observed only for mothers with lower levels of harm-avoidance and higher levels of perceived social support. Importantly, the results were not better explained by prematurity, pregnancy complications, exposure to drugs, alcohol or environmental toxins.

**Conclusions:**

The findings support multidimensional theoretical models of prenatal stress. Although both objective stressors and subjectively measured distress predict birthweight, they should be considered distinct and cumulative components of stress. This study further highlights that jointly considering risk factors and protective factors in pregnancy improves the ability to predict birthweight.

## Introduction

**Fig 1:**
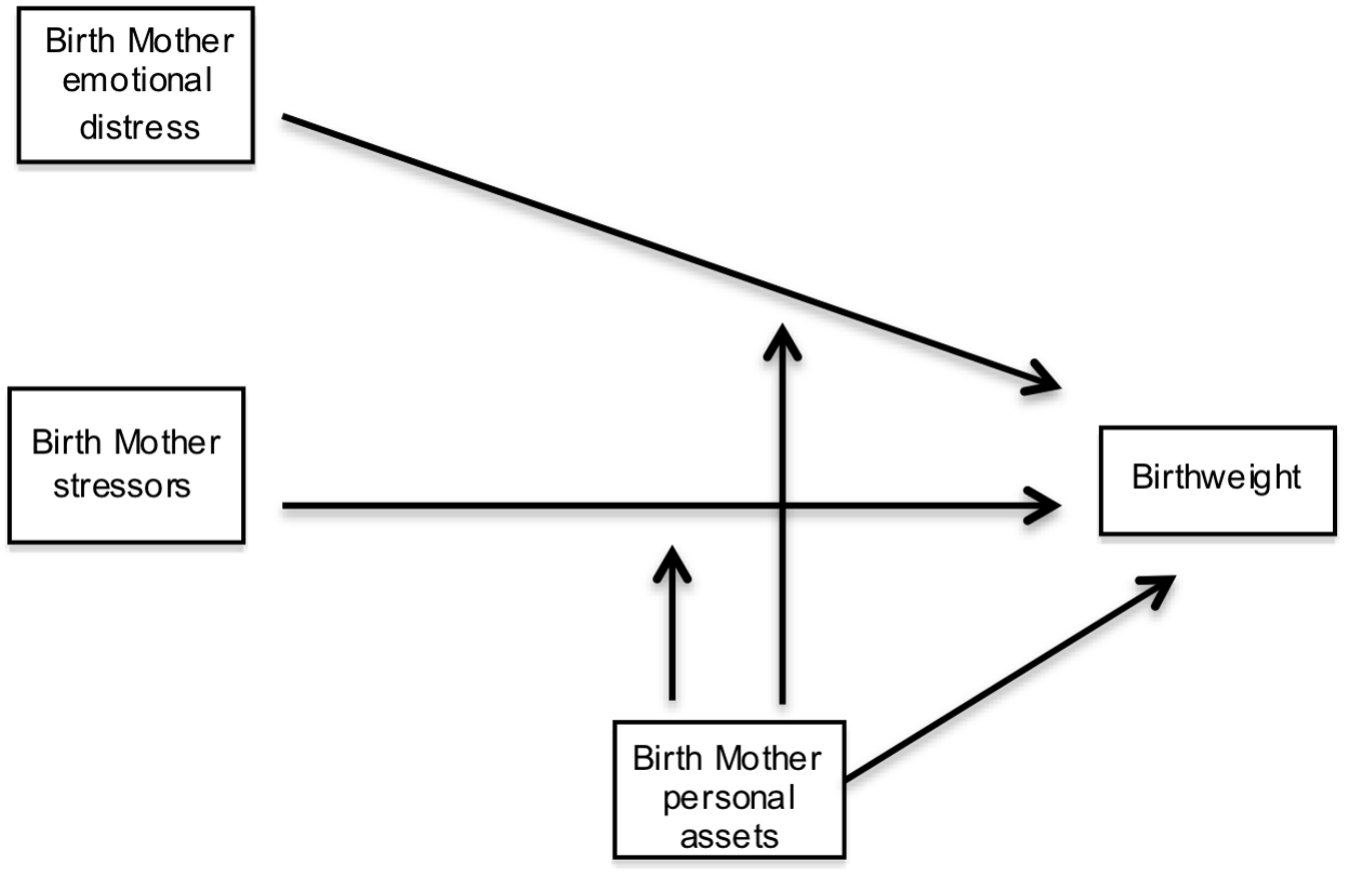
Model of multiple factors contributing to prenatal stress, maternal characteristics and birthweight.

A core finding of developmental science is that early life experiences predict subsequent child development. Development is most rapid during the prenatal period [1] and identifying modifiable environmental influences during this period may be important for reducing later problems [2]. Exposure to maternal stress in the womb predicts poor outcomes for children across a range of developmental measures [3–6]. These outcomes begin with effects on fetal growth and birthweight. Many studies of these effects use a single measure of stress, limiting our understanding of the combination of multiple types of stress [7–9]. Additionally, the causal relationship between maternal stress and fetal development is difficult to establish [10] when confounding contributions of other intrauterine environmental exposures—such as toxins and substances of abuse—are not considered [11]. Moreover, while characteristics of the pregnant mother have been studied based on their potential protective effects from exposure to maternal stress in utero in relation to postnatal outcomes [12], the interaction of maternal characteristics with prenatal stress has received much less attention for its effect on fetal growth and birthweight [13]. The present study tests a model combining effects of external stressors, of distress reactions and of maternal personal characteristics on birthweight while controlling for other pre- and peri-natal environmental influences (Fig 1).

### Measuring maternal stress in pregnancy: a multidimensional model

Stress is often defined as a threat to physiological and/or psychological homeostasis. [14]. Accordingly, stressors are stimuli that can trigger physiological, emotional, and/or behavioral reactions of distress when perceived as exceeding available resources [15]. An inidvdual’s response to threat reflects the balance between apparent demands and available resources [16].

During postnatal development, Belsky [17] proposed that external stressors do not predict parenting behavior as well as parents’ personal characteristics do, including personality and perceptions of stressors and support. Indeed, parental personality predicts the relationship between stress and positive parenting behaviors such as sensitivity to child cues, responsiveness and affective support [18, 19], which are also related to child outcomes [20, 21]. *In utero*, maternal personality and stress impact fetal health through a complex set of physiologic reactions that combine to shape the intrauterine environment [22]. A mother’s ability to moderate her physiologic response to stress has been postulated to protect maternal and fetal health (e.g., [23]. Maternal factors that impact her perception of or ability to manage stressors are of paramount importance [9].

### Measuring associations between maternal stress and infant birthweight

Birthweight is influenced by many factors but is, in part, a *direct* consequence of maternal stress. With suitable control of gestation time, birthweight is a good indicator of fetal growth. Maternal mood and chronic and acute stressors in pregnancy are associated with increased risk of low birthweight [24, 25] even after controlling for confounding psychosocial, behavioral and physiological factors [26, 27]. Mothers’ stress may also be *indirectly* correlated with birthweight via other health behaviors during pregnancy, including nutrition and substance use [28–30].

In turn, low birthweight is associated with multiple domains of a child’s postnatal health, including higher prevalence of childhood ADHD, depression, anxiety and schizophrenia [31–35]. Even within the normal range, birthweight is inversely proportional to an array of indicators of childhood adaptation such as learning, behavioral regulation and autism [36].

### Measuring prenatal maternal stress

#### Distress

Many studies investigating prenatal stress and birth outcomes have used *maternal mood* during pregnancy as an indicator of distress [37, 38]. Other distress reactions also predict fetal stress and birthweight, such as *worry* specific to the pregnancy experience [39, 40].

#### Stressors

Distress-variables used to index fetal stress-exposure do not overlap entirely with exposure to external stressors [14]. Common prenatal *negative life events* [41], *objectively measured large-scale stressors* such as war and natural disasters [42–44], and psychological and physical *relational conflict* [45–47] all predict reduced birthweight. In addition, low *socio-economic status* (SES) is typically associated with multiple stressors and consistently predicts low birthweight even when controlling for the impact of mothers’ negative health related behaviors [48]. Finally, based on the documented importance of grandparents in supporting young parents in pregnancy and infant care (e.g. [49], maternal grandparents’ disruptive and unstable behaviors may be considered a specific form of *chronic family stressor* that acts on fetal growth by way of mother’s response.

#### Maternal characteristics

Maternal characteristics may moderate the association between stressor exposure and post partum child outcomes [16]. Indeed, Guardino and Dunkel Schetter [9] suggest that maternal *personality* or *character* and its interaction with stress should be investigated further, as it predicts stress management strategies and behaviors, which, in turn, predict levels of general and pregnancy-specific distress [50]. There are associations between maternal *self-esteem* and other *character* traits—such as self-efficacy and dispositional optimism—on birthweight [13, 51]. *Perceived social support* is a core factor during pregnancy [52] and inversely related to birthweight in high- and low-risk populations [53–57]. Recent literature has established that perception of social support reflects the characteristics of an individual as much as, or more than, the coincidental availability of supportive others [58]; thus, and it is not suprising that it is as heritable as other measures of personality [59].

### The present study

This study will test a conceptual model of the association between the maternal stress experience and child birthweight (Fig 1). Relations among emotional distress (*mood* and *material worry*), maternal stressors (*negative life events*, *relational conflict*, *SES*, and *chronic family stress*), maternal characteristics (*self-esteem*, *temperament*, and *perceived social support*), and birthweight will be tested. We predict that distinct stressor and distress variables will represent multifactorial risk and correlate independently with birthweight. Personal characteristics are proposed to moderate the association between stress and birthweight, such that mothers high in purportedly protective personal characteristics would have higher birthweight children under stressful circumstances than mothers low in such traits. Indices of distress such as depressive symptoms already represent, in part, an adverse response to external stressors. In our conception, these are a *result of* a relative paucity of protective factors and their influence on fetal growth is unlikely to be further moderated by maternal characteristics.

The present report uses data collected in a long-term prospective adoption study designed to closely examine multiple psychosocial influences on the development of both psychopathology and social competence in children and adolescents. Current state-of-the-art studies of influences on birth weight use data collected prospectively during pregnancy, but an adoption study is ethically constrained from collecting data before the adoption has occurred. However, in other respects our cohort is well suited. Some birth mothers contemplating placing their child, live out their pregnacies in high levels of distress, so there is substantial variability of this dimension. This variability is also true of their relationships and social support. During pregnancy our measurement captured multiple aspects of the birth mother’s psychological and social environment, as well as her use of substances and her exposure to toxins that are critical controls in birthweight studies. Although necessarily retrospective, our assessment of prenatal stress is minimally affected by birth mother’s current relationship with or observations of the child she has placed. Lastly, children adopted at birth, who may be a special population in the impact of stress, have likely been absent from or a small minority of most studies on prenatal stress. Among other aims, this study seeks to test whether previous findings on prenatal stress and birthweight generalize to a sample of biological mothers and the children they place for adoption.

## Method

### Participants

The Early Growth and Development Study (EGDS) is a longitudinal prospective adoption study consisting of domestic US adoption triads (adoptive children, birth parents and adoptive parents) studied at intervals from birth through child age 7 or 11 [60]. The study was designed to investigate specific environmental and genetic effects on child development and the sample is representative of domestic adoptions, recruited from adoption agencies across the United States. Birth mothers were predominantly caucasian (68.8%) and african-americanAfrican-American (13.6%), and the large majority (71.9%) had obtained a high-school diploma or less at the time of adoption. Of the initial sample of *N* = 877 birth mothers, the present study included all birth mothers (BM *n* = 403) for which the full set of variables was available (availabity for each variable from 877: Birthweight 777; Pregnancy risk index 877; Openness 725; Negative life events 838; Socioeconomic status 862; Chronic family stress 678; Relational conflict 705; Mood 844; Material worry 823; Maternal characteristics 868) [60] (Table 1). The initial EDGS sample did not differ signficantly from the final sample used here on any study variables, with outliers removed as described in results. The collection of data from birth mothers at the first assessment was conducted independently of whether the adoptive family was ultimately recruited. Thus, the sample presented here includes participants not enrolled in subsequent assessments reported elsewhere. Recruitment took place 1–3 months after the adoption process had been legally concluded. The median child age at adoption was 2 days and most children were placed for adoption within the first week of life (Table 1). 56.3% of the children were male. Additional details on the EGDS study recruitment procedures, sample, assessment methods, and approvals are available elsewhere [60].

**Table 1:** Sample demographics.

Variable	Final sample (N = 403)
M child age in days at child adoption (SD)	6.2 (12.45)
Race (%)	
Caucasian	75.3
African-American	8.2
Hispanic	7.8
Multi-ethnic	4.3
Other/not reported	4.4
Median level of education	High school degree
Less than a high school degree (%)	19.6
G.E.D. degree (%)	15.7
High school degree (%)	39.0
Trade school (%)	11.8
2-year college or university (%)	6.9
4-year college or university (%)	6.1
Graduate program (%)	0.8
Median household income ($)	15000–25000

### Procedure

Only information from the initial assessment of the birth mothers was used, collected retrospectively at 4 months postpartum. Private, in-person interviews consisted of both interviewer-administered questions and computer-assisted questionnaires. Questionnaires were designed to maximize the likelihood of participants providing information relative to the pregnancy and not the post-partum period using the robustly validated life-history interview technique [61, 62].

### Measures

#### Prenatal stress

**Emotional distress**: *Anxious and depressed mood* during pregnancy was quantified using a representative subset of items from the Beck Anxiety Inventory and the Beck Depression Inventory [63, 64]. The subset did not include somatic symptoms of depression and anxiety that can be confounded by normal symptoms of pregnancy but included core questions [briefly: worrying, pounding heart, terrified feeling, lost interest or sad feeling, feelings of failure, feelings compared to others, ability to work and guilty feelings]. Based on their intercorrelation (*r*_*(489)*_ = .43, *p* < .001), the 9 items were combined into an aggregate *mood* scale (Cronbach’s *α* < .9) to limit multicolinearity and maximize power.

*Material worry*. This is a measure of non-clinical distress related to mothers’ financial satisfaction and concept of herself as a provider of care to children. Such measures of financial satisfaction are relatively independent from actual financial means [65].

Items from the Family Demographics Scale [65] were combined to yield aggregate scores indexing different aspects of worry about material resources. Specifically, on a scale from 1–5, participants indicated whether they felt able to secure a fitting home, food and clothing, whether they had difficulty paying bills, and the extent to which they were forced to make significant cuts in spending in the last year. Scores on the three scales were standardized and summated yielding an overall scale of material worry (Cronbach’s α = .66-.87).

**Stressors**: *The negative life events (NLE)* scale sums the presence of 31 experiences rated as having the potential to disturb everyday-life routines and provoke distress-reactions during the pregnancy [41].

The 8-item *relational conflict* scale was established for the present study and indexes physically or psychologically abusive or negatively charged interactions with family members, close friends and life partner relating specifically to the pregnancy and adoption process (α = .66). For instance, birth mothers were asked to rate the emotional response of others to the news of the pregnancy and the frequency of violent behaviors. Based on its moderate and negative correlation with *perceived social support* (*r*_(489)_ = -.47, *p* < .001)—likely due to items inquiring about negative and positive aspects of the *same* relationships—only 4 items were retained namely those inquiring specifically about negative reactions to the pregnancy and adoption plan.

*Chronic family stress*. Birth mothers reported on the presence of marital conflict, alcohol and substance abuse, previous treatment for psychiatric symptoms, aggression and delinquent behavior in their own parents. The scale is adapted from the *Family History—Research Diagnostic Criteria* [66]. Here, items relating to each grandparent separately were summed (α = .81).

*Socioeconomic status* (*SES*). Three factors were combined: educational attainment, household income, and interviewer ratings of neighborhood safety. Regarding the latter, *interviewers* were asked to rate the accuracy of statements such as “This is a safe neighborhood for elementary school age kids to play on the sidewalk unattended” or “There were obvious signs of delinquent activities in the neighborhood”. Scores on each scale were standardized and summed (Cronbach’s α = .89).

#### Maternal characteristics

*Self-esteem* was quantified using the Harter Adult Self-Perception Profile [67]. Scores on six subscales (global self-worth, sociability, nurturance, adequate as a provider, intimate relationships, and sense of humor) were standardized and summated to yield an overall score (Cronbach’s α = .77).

*Temperament/Character* was indexed using the Temperament Character Inventory (TCI) [68]. Scores on personality traits associated with poor adaptation (Novelty seeking and Harm avoidance) were reverse-coded and then summed with the remaining five (Reward Dependence, Persistence, Self-directedness, Self-transcendence, Cooperativeness) yielding an overall protective temperament/character index (α = .66)

*Perceived social support* was indexed using the satisfaction with the support received or available from intimate relationships, friends, and the community. This construct combined standardized scores on two subscales of the General Life Satisfaction questionnaire (Crnic et al., 1983), and 4 items from the Adoption Process Scale developed for EGDS [69] (Cronbach’s α = .88).

To maximize power [70] *self-esteem*, *temperament/character*, and *perceived social support* (*r*_(489)_ = .26 –.53, *p* < .01), scores were combined into one aggregate *protective maternal characteristics* scale for initial analyses (Cronbach’s α = .67).

In addition to adequate reliability, scores on all scales are normally distributed and have satisfactory range. The final birth mother variables included in the analyses were converted to *z*-scores and the interaction terms between stressors and maternal characteristics were created using standardized scores. Table 2 summarizes the inter-correlations among the final set of variables included in the regression analyses.

**Table 2:** Table of inter-correlations final set of study variables of birth mothers and birthweight (*N* = 403).

				Distress		Stressors				
Variable	BW	PRI	Open	Mood	MW	NLE	SES	CFS	RC	Maternal Characteristics
BW	1									
PRI	-.03	1								
Openness	.13**	-.02	1							
Mood	-.11**	.27**	-.04	1						
MW	-.19**	.15**	-.15**	.23**	1					
NLE	-.04	.21**	.04	.26**	.23**	1				
SES	.19**	-.04	.12*	.02	-.23**	-.10*	1			
CFS	.11*	-.07	.05	.20**	.06	.06	.14**	1		
RC	.02	-.16**	.13**	.18**	.17**	.25**	-.17**	-.05*	1	
Maternal char-acteristics	.14**	-.06	.16**	-.38**	-.29**	-.17**	.17**	-.02	.06	1

*Note*. BW = Birthweight, PRI = Pregnancy Risk Index, NLE = Negative Life Events, SES = Socio-economic Status, CFS = Chronic Family Stress, RC = Relational Conflict, MW = Material Worry.

*p < .05

**p < .01.

#### Child outcome measure: birthweight

The dependent variable in the present study is child birth-weight as reported by the birth mother. Medical records containing birthweight were available for approximately 60% of the sample and correlated highly with self-report (*r* = .92, *p* < .001), consistent with previous reports [71, 72].

Gestational age was available through medical records for 60% of our sample All birth mothers in our sample were asked to report whether or not their child was full term (defined for them as 37–41 weeks gestational age). To test the possibly confounding effect of prematurity on the association between stress, maternal characteristics and birth weight, we conducted a secondary regression analysis including only birth mother reported full-term infants.

### Covariates

Using the Life History Calendar method [61] to maximize response accuracy, birth mothers reported on their exposure at specific time points during pregnancy to environmental toxins (radiation, X-rays, lead, and chemical toxins), cigarette smoking, alcohol, and illicit substances (alcohol, marijuana, cocaine, hallucinogens, amphetamines, heroin, prescription painkillers (used illegally), inhalants, sedatives, and tranquilizers). This information along with pregnancy-related medical complications (maternal age, prenatal care, weight loss, weight gain, nausea, pre-eclampsia symptoms, HIV/AIDS, infections, fetal stillness) were summed using the Pregnancy Risk Index and entered first in the model below to control for confounding influences on birthweight [73].

To examine the potential effect of adoption openness, birth mothers’ and adoptive parents’ reports of openness, the level of contact between the birth and adoptive parents (five scales ranging from 1 [*never*] to 5 [*daily*]), and the extent of knowledge about each other (six scales ranging from 1 [*a lot*] to 4 [*nothing*]) were aggregated and included as a covariate.

### Data analytic strategy

We sought to test a parsimonious model relating the multiple indicators of distress, stressors, and maternal characteristics to birthweight, hypothesizing that the groups of variables would each add to the effect. A moderated hierarchical regression was performed using SPSS (IBM) to estimate the amount of variance in child birthweight explained by stressor and distress variables separately and by the interaction among stressors and maternal characteristics. The Adoption Openness Scales and Pregnancy Risk Index covariate were entered in the first two steps as a control. The distress variables were then entered in a separate step, followed by the block of stressor variables to investigate their effect over and above that of distress scores. That is, we sought in this fourth step to understand whether there were direct effects of stressors that were not mediated by measures of subjective distress. Maternal characteristics were then added. The interaction between characteristics and the stressor variables was entered as a last step. To ascertain the statistical significance of each interaction, each product term was also entered separately in a second regression model before regions of significance analyses were conducted to interpret statistically significant interactions.

## Results

Results of the descriptive analyses for all birth mother variables are reported in Table 3. Outliers in the self-reported birthweight distribution were visually identified and newborns larger than 10.74 lbs *(n* = 3) and smaller than 4.30 lbs *(n* = 8) were not included in the analyses. The final sample size reflects the number of BMs (*n* = 403) without missing data on any of the selected variables excluding the identified outliers.

**Table 3:** Descriptive statistics for Birth mothers (*N* = 403).

Variable	*M*	*SD*	Skewness	Kurtosis
Birthweight (lbs)	7.27	1.11	0.03	-0.22
Openness^a^	0.00	0.85	-0.02	-0.08
Pregnancy Risk Index	8.48	6.20	1.33	2.60
Anxiety	4.28	4.24	0.36	- 1.22
Depression	7.73	5.14	-0.06	- 0.73
Material worry^a^	-0.01	2.30	0.14	- 0.59
Relational conflict^a^	0.62	0.31	-0.39	-0.67
Negative life events	6.38	3.80	0.56	-0.01
Socio-Economic Status^a^	0.01	1.00	0.83	2.79
Chronic Family Stress^a^	0.23	1.53	0.14	- 0.71
Temp/character^a^	0.18	3.94	-0.38	- 0.04
Self-esteem^a^	-0.02	3.92	-0.25	- 0.50
Social support^a^	0.08	3.16	-0.84	1.16

*Note*. All final scales included in the study were standardized prior to data analysis

(*M* = 0, *SD* = 1).

^a^ Based on standardized scores

BW = Birthweight, PRI = Pregnancy Risk Index, NLE = Negative life events, SES = Socio-economic Status, CFS = Chronic Family Stress, RC = Relational conflict, MW = Material Worry

### Results of overall regression model

The final model contained six blocks (Table 4). First, results indicated that Adoption Openness accounted for 1.8% of variance in birthweight (*F*_(1, 401)_ = 7.204, *p* = .008). The Pregnancy Risk Index did not account for any variance in birth weight (*F*_(1, 400)_ = 0.323, *p* = .570). In the third block, results indicate that distress measures accounted significantly for 3.2% of the variance in birthweight (*F*_(2, 398)_ = 6.782, *p* = .001). The four stressors were subsequently entered into the regression equation and the significant change in variance accounted for by all of these was equal to 4.9% (*F*_(4, 394)_ = 5.339, *p* < .001).

**Table 4:** Summary of hierarchical regression predicting child birthweight from birth mother distress, stressors and protective maternal characteristics.

	Birth mothers (*N* = 403)		
Predictor	*B*	*R* ^*2*^ *Δ*	*FΔ*
Openness	0.15**	.018	7.204
Pregnancy Risk Index	-0.03	.001	.323
Distress		.032	6.782**
Mood	-0.08		
MW	-.018**		
Stressors		.049	5.339**
NLE	-0.01		
SES	0.20**		
CFS	0.19**		
RC	0.02		
Maternal characteristics	0.03	.001	.270
Maternal characteristics Interaction		.031	3.413**
NLE*Maternal characteristics	0.09		
SES* Maternal characteristics	.14*		
CFS*Maternal characteristics	-0.08		
RC*Maternal characteristics	-0.14^α^		
Total *R* ^*2*^ (adjusted)	.131** (.102**)		

*Note*. All variables (except interaction terms) were standardized before being entered into the regression model. MW = Material Worry, NLE = Negative life events, SES = Socio-economic Status, CFS = Chronic Family Stress, RC = Relational conflict.

^α^ *p* < .1

*p < .05

**p < .01

The fifth step contained only *maternal characteristics* and did not account for a signficant change in variance (*F*_(1, 393)_ = 0.270, *p* = .604). The final step, which included the four product terms between *stressors* and *maternal characteristics*, was associated with an additional 3.1% of variance (*F*_(4, 389)_ = 3.413, *p* = .009). The hierarchical regression model as a whole accounted for 13.1% of the observed variance in birthweight.

### Secondary Analyses

**Fig 2:**
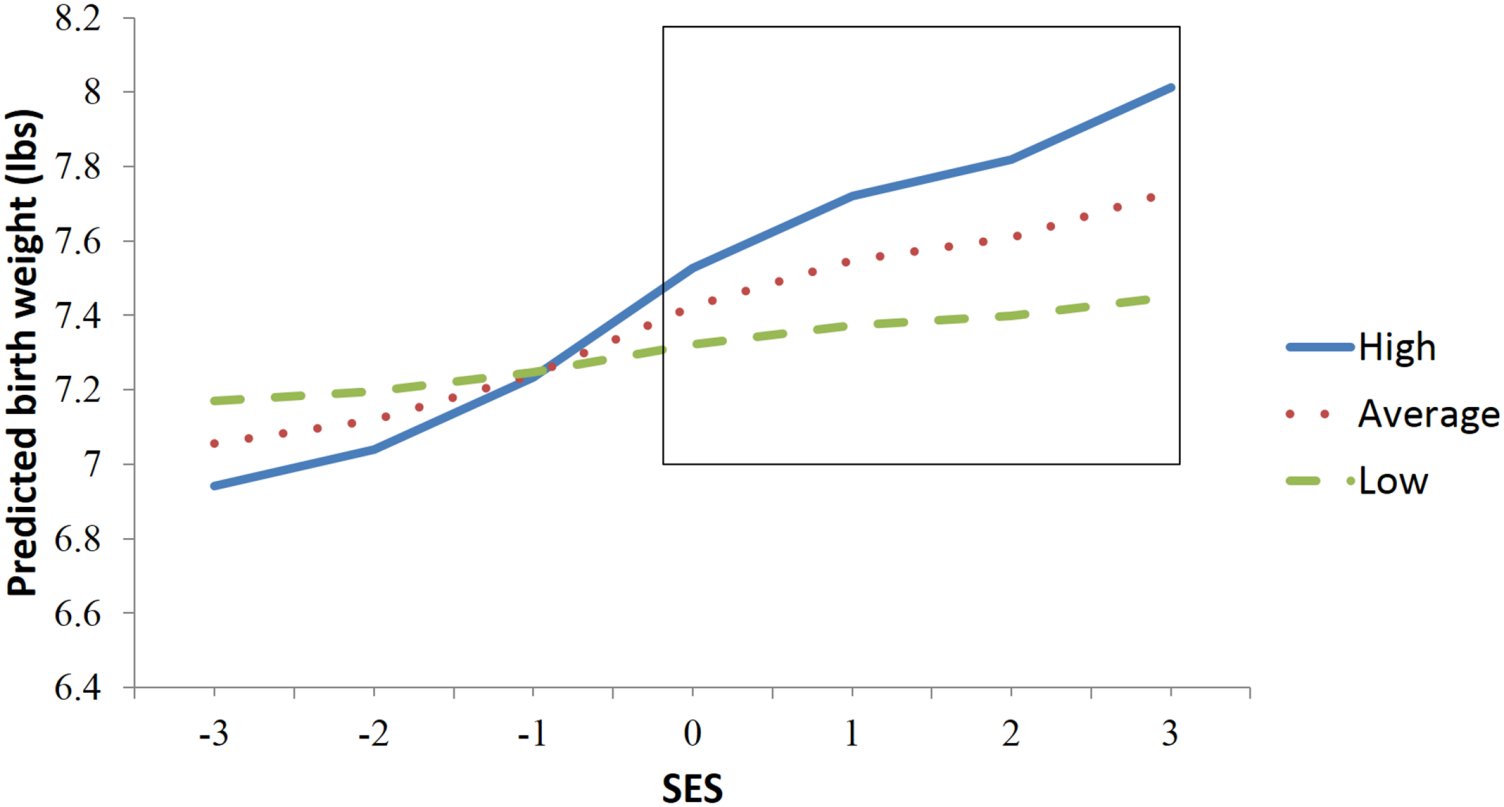
Interaction between socio-economic status and maternal characteristics in predicting birthweight plotted at the mean, 1 SD above and 1SD below the mean on maternal characteristics scale.

Examining each maternal characteristic/stressor interaction separately revealed that only SES (*B* = 0.12, *t*_(402)_ = 2.16, *p* = .031) had a differential impact on birthweight as a function of maternal characteristics. Other interactions were not significant (NLE: *B* = 0.06, *t*_(402)_ = 1.97, *p* = .285, RC: *B* = -0.10, *t*_(402)_ = -1.95, *p* = .052 CFS: *B* = -0.05, *t*_(402)_ = -0.88, *p* = .379). SES was associated with birthweight only for mothers with average (*B* = 0.11, *t*_(402)_ = 3.68, *p* < .001) and above average maternal characteristics (*B* = 0.17, *t*_(402)_ = 3.95, *p* < .001) not for mothers with low scores on personal characteristics (Fig 2). A region of significance analysis was conducted [74] and identified that the significantly discriminable differences between the slopes occurred above -0.19 *SD* from the mean of the SES distribution. Mothers with high levels of protective characteristics at these high and low levels of SES did not differ significantly on any other measured variables.

Post-hoc analyses suggested mothers’ perceived social support scores *(R* ^*2*^ = 13.6%, *F*_(4, 389)_ = 4.07, *p* = .003) and the TCI subscale Harm Avoidance (*R* ^*2*^ = 12.1%, *F*_(4, 379)_ = 2.35, *p* = .05) drove the interaction with the stressor variables as indices of *maternal characteristics*.

To control for the possible effect of prematurity on birth weight in the present study, the above analysis was repeated with only infants reported by birth mothers as being born at full term (≥37 weeks of gestation) (Table 5). Despite the relative decrease in statistical power related to sample size reduction, the results of the regression model using the sample of 356 full-term infants remained broadly similar with a slight increase in variance explained from 13.1 to 15.0% (*F*_(4, 342)_ = 3.08, *p* = .016).

**Table 5:** Summary of hierarchical regression predicting child birthweight from birth mother distress, stressors and protective maternal characteristics in full term infants only.

	Full term infants (N = 356)		
Predictor	*B*	*R* ^*2*^ *Δ*	*FΔ*
Openness	0.12*	0.012	4.481*
Pregnancy Risk Index	-0.03	0.001	0.369
Distress		0.017	3.037*
Mood	-0.05		
MW	-0.12*		
Stressors		.085	8.326**
NLE	0.02		
SES	0.28**		
CFS	0.21**		
RC	0.03		
Maternal characteristics	0.08	.004	1.576
Maternal characteristics Interaction		.031	3.076*
NLE*Maternal characteristics	0.10^α^		
SES* Maternal characteristics	0.11^α^		
CFS*Maternal characteristics	-0.10^α^		
RC*Maternal characteristics	-0.13*		
Total *R* ^*2*^ (adjusted)	.150* (11.8)		

*Note*. All variables (except interaction terms) were standardized before being entered into the regression model. MW = Material Worry, NLE = Negative life events, SES = Socio-economic Status, CFS = Chronic Family Stress, RC = Relational conflict.

^α^ *p* < .1

*p < .05

**p < .01

## Discussion

We found small but unique associations of prenatal maternal distress and maternal stressors with fetal development. We also found that maternal characteristics interacted with stressors in predicting birthweight, suggesting the importance of maternal adaptation. Broadly, the results here demonstrated that a multifactorial model of stress with multiple indicators was most accurate at predicting birthweight. Both birthmother distress and stressor variables during pregnancy were found to predict variance in birthweight. Taken together, the observed findings and extant theoretical framework support that a reliable and sensitive operationalization of stress should reflect its multidimensional nature. Limiting measures of stress in pregnancy to mood or life events may underestimate the effect on child development, not capturing overlapping yet distinct components [14]. In line with our hypotheses, the inclusion of stressor variables was associated with more than a twofold increase in predictive power of the regression model. Approximately 13% of birthweight variance was predicted by multiple measures of prenatal stress and characteristics with only minor differences observed when only full term infants were included.

### Interaction of stressors and maternal personal characteristics

Protective personal characteristics measured alongside multiple measures of distress and stressor exposure sets the present study apart [9). Specifically, we hypothesized that measures of maternal self-esteem, temperament/character and perception of social support would moderate the impact of stressors on birthweight, as these factors have been found to predict the appraisal of and psychological adaptation to such experiences [15]. In line with the hypothesized model, no direct effect of these maternal characteristics on birthweight was observed. Such characteristics thus only predicted birthweight differences under circumstances with different levels of stress [14]. As a whole, the interaction between stressors and maternal maternal characteristics while small, increased the amount of variance of birthweight accounted for by 30%. In addition to the observed main effect, findings indicated that the ability of SES to predict birthweight was also contingent upon maternal characteristics. This conclusion was substantiated by the fact that mothers with high levels of personal characteristics in high and low socioeconomic status did not differ on any other measured variables. In terms of stress measurement, the observed results thus imply that both the full effect of external risk factors (stressors) and maternal characteristics is seen if they are jointly considered.

Low SES has been construed as a broad risk-marker for low birthweight [37, 75] and we expected to find this association attenuated in the presence of personal characteristics. The region of significance analysis demonstrated instead that *increased* levels of education, income, and neighborhood safety were associated with fetal weight gain only for mothers at average or above average levels of such characteristics. It is noteworthy that an average level of SES in the current sample is lower than the general population. However, in addition to operating as a buffer against adversity at lower levels of SES, these chracteristics may also have enhanced mothers’ capacity to gain from favorable circumstances. This result is consistent with well-validated theoretical frameworks of development [76, 77].

In summary, the multiple regression analyses demonstrated that distress and stressor variables both overlapped and interacted yet were distinct components of stress [38, 56, 78, 79]. The findings further suggest that jointly considering risk factors and protective factors in pregnancy improves the ability to predict child developmental outcomes [9]. In the context of prenatal stress, mothers should not be considered passive respondents to external influences. Maternal characteristics may actively influence fetal health and development. The present study also suggests that the observed findings were not better explained by other environmental risk factors, as controlled for by covariate inclusion of exposure to a range of known pregnancy risk factors which had insignificant effects on outcomes as previously discussed [72]. The results were replicated in a subsample of fullterm infants, ascertaining that gestational age was not responsible for the observed findings.

However, the present study does not address the causal mechanisms underlying the association between maternal psychological states and child development. For example, particularly in this cohort of birth mothers who decided to place their child for adoption at birth, stress may be *indirectly* correlated with birthweight via other health behaviors during pregnancy [2, 28–30]. Indeed, although weight at birth can be construed as an indicator of uterine environmental adversity, low birthweight per se might not be causally related to subsequent poor outcomes [80, 81] and may differentially affect the type of subsequent outcomes [80]. Importantly, there are likely also physiologically relevant effects of prenatal stress not indexed by birthweight [80]. Further research on the specific mechanisms underlying the transduction of stress from mother to fetus is necessary to elucidate these links.

Additionally, the present study cannot fully exclude biases introduced with retrospective reports despite the lack of influence of postnatal parenting experiences and steps taken to assure the accuracy of self-reported data. Furthermore, factors relating specifically to adoption such as the rationale behind placing the child, the timing of the adoption plan, and compensation to the birth mother could not be investigated in the present study. Other potential limitations include the use of SES as a measure of external stress despite the possible contribution of inherent maternal characteristics to this measure.

Despite these concerns, the current study provides evidence supporting the use of multiple indicators of distress and stressors to measure the integrated effects of stress in pregnancy. More important, the present findings clarify that both stressors and distress contribute to birthweight and emphasize the importance of considering maternal characteristics as buffers against those adversities in fetal development.

## Supporting Information

S1 Table Complete model summary of hierarchical regression predicting child birthweight from birth mother distress, stressors and protective maternal characteristics. (DOCX)

S2 Table ANOVA for main hierarchical regression model analysis. (DOCX)

S3 Table Complete multiple regression coefficients from main model. (DOCX)
