# Correction: Expectant Mothers Maximizing Opportunities: Maternal Characteristics Moderate Multifactorial Prenatal Stress in the Prediction of Birth Weight in a Sample of Children Adopted at Birth

**DOI:** 10.1371/journal.pone.0230595

**Published:** 2020-03-12

**Authors:** Line Brotnow, David Reiss, Carla S. Stover, Jody Ganiban, Leslie D. Leve, Jenae M. Neiderhiser, Daniel S. Shaw, Hanna E. Stevens

The following information is missing from the Funding statement: This project was supported by the Klingenstein Third Generation Foundation Fellowship and CTSA Grant Number UL1 TR001863 from the National Center for Advancing Translational Science (NCATS).

## References

[1] Brotnow L, Reiss D, Stover CS, Ganiban J, Leve LD, Neiderhiser JM, et al (2015) Expectant Mothers Maximizing Opportunities: Maternal Characteristics Moderate Multifactorial Prenatal Stress in the Prediction of Birth Weight in a Sample of Children Adopted at Birth. PLoS ONE 10(11): e0141881 10.1371/journal.pone.0141881 26544958 PMC4636431

