# Supplementary material for: Expectant Mothers Maximizing Opportunities: Maternal Characteristics Moderate Multifactorial Prenatal Stress in the Prediction of Birth Weight in a Sample of Children Adopted at Birth
Source: PLoS One. 2015 Nov 6;10(11):e0141881. doi: 10.1371/journal.pone.0141881 (PMC4636431; doi:10.1371/journal.pone.0141881)
Supplement: S1 Table — (DOCX) [file pone.0141881.s001.docx]

| **S1 Table. Complete model summary of hierarchical regression predicting child birthweight from birth mother distress, stressors and protective maternal characteristics.** | | | | | | | | | |
| --- | --- | --- | --- | --- | --- | --- | --- | --- | --- |
| Model | R | R Square | Adjusted R Square | Std. Error of the Estimate | Change Statistics | | | | |
|  |  |  |  |  | R Square Change | F Change | df1 | df2 | Sig. F Change |
| 1 | .133^a^ | .018 | .015 | 1.09903 | .018 | 7.204 | 1 | 401 | .008 |
| 2 | .136^b^ | .018 | .014 | 1.09996 | .001 | .323 | 1 | 400 | .570 |
| 3 | .225^c^ | .051 | .041 | 1.08440 | .032 | 6.782 | 2 | 398 | .001 |
| 4 | .316^d^ | .100 | .081 | 1.06150 | .049 | 5.339 | 4 | 394 | .000 |
| 5 | .317^e^ | .100 | .080 | 1.06249 | .001 | .270 | 1 | 393 | .604 |
| 6 | .362^f^ | .131 | .102 | 1.04968 | .031 | 3.413 | 4 | 389 | .009 |
| a. Predictors: (Constant), Openness | | | | | | | | | |
| b. Predictors: (Constant), Openness, PRI | | | | | | | | | |
| c. Predictors: (Constant), Openness, PRI, MW, mood, | | | | | | | | | |
| d. Predictors: (Constant), Openness, PRI, MW, mood, RC, SES, CFS, NLE, | | | | | | | | | |
| e. Predictors: (Constant), Openness, PRI, MW, mood, RC, SES, CFS, NLE, maternal characteristics | | | | | | | | | |
| f. Predictors: (Constant), Openness, PRI, MW, mood, RC, SES, CFS, NLE, maternal characteristics, NLE* mat char, SES*mat char, RC*mat char, CFS*mat char  Note: PRI = Pregnancy Risk Index, NLE = Negative Life Events, SES = Socio-economic Status, CFS = Chronic Family Stress, RC = Relational Conflict, MW = Material Worry. | | | | | | | | | |
