# Supplementary material for: Expectant Mothers Maximizing Opportunities: Maternal Characteristics Moderate Multifactorial Prenatal Stress in the Prediction of Birth Weight in a Sample of Children Adopted at Birth
Source: PLoS One. 2015 Nov 6;10(11):e0141881. doi: 10.1371/journal.pone.0141881 (PMC4636431; doi:10.1371/journal.pone.0141881)
Supplement: S2 Table — (DOCX) [file pone.0141881.s002.docx]

| **S2 Table. ANOVA for main hierarchical regression model analysis** | | | | | | |  |
| --- | --- | --- | --- | --- | --- | --- | --- |
| Model | | Sum of Squares | df | Mean Square | F | Sig. |  |
| 1 | Regression | 8.701 | 1 | 8.701 | 7.204 | .008^b^ |  |
|  | Residual | 484.358 | 401 | 1.208 |  |  |  |
|  | Total | 493.059 | 402 |  |  |  |  |
| 2 | Regression | 9.091 | 2 | 4.546 | 3.757 | .024^c^ |  |
|  | Residual | 483.968 | 400 | 1.210 |  |  |  |
|  | Total | 493.059 | 402 |  |  |  |  |
| 3 | Regression | 25.042 | 4 | 6.261 | 5.324 | .000^d^ |  |
|  | Residual | 468.017 | 398 | 1.176 |  |  |  |
|  | Total | 493.059 | 402 |  |  |  |  |
| 4 | Regression | 49.106 | 8 | 6.138 | 5.448 | .000^e^ |  |
|  | Residual | 443.953 | 394 | 1.127 |  |  |  |
|  | Total | 493.059 | 402 |  |  |  |  |
| 5 | Regression | 49.411 | 9 | 5.490 | 4.863 | .000^f^ |  |
|  | Residual | 443.649 | 393 | 1.129 |  |  |  |
|  | Total | 493.059 | 402 |  |  |  |  |
| 6 | Regression | 64.451 | 13 | 4.958 | 4.500 | .000^g^ |  |
|  | Residual | 428.608 | 389 | 1.102 |  |  |  |
|  | Total | 493.059 | 402 |  |  |  |  |
| a. Dependent Variable: Birthweight (lbs) | | | | | | | |
| b. Predictors: (Constant), Openness, | | | | | | | |
| c. Predictors: (Constant), Openness, PRI, | | | | | | | |
| d. Predictors: (Constant), Openness, PRI, MW, mood, | | | | | | | |
| e. Predictors: (Constant), Openness, PRI, MW, mood, RC, SES, CFS, NLE, | | | | | | | |
| f. Predictors: (Constant), Openness, PRI, MW, mood, RC, SES, CFS, NLE, maternal characteristics, | | | | | | | |
| g. Predictors: (Constant), Openness, PRI, MW, mood, RC, SES, CFS, NLE, maternal characteristics, NLE* mat char, SES*mat char, RC*mat char, CFS*mat char | | | | | | | |

Note: PRI = Pregnancy Risk Index, NLE = Negative Life Events, SES = Socio-economic Status, CFS = Chronic Family Stress, RC = Relational Conflict, MW = Material Worry.
