# Supplementary material for: Expectant Mothers Maximizing Opportunities: Maternal Characteristics Moderate Multifactorial Prenatal Stress in the Prediction of Birth Weight in a Sample of Children Adopted at Birth
Source: PLoS One. 2015 Nov 6;10(11):e0141881. doi: 10.1371/journal.pone.0141881 (PMC4636431; doi:10.1371/journal.pone.0141881)
Supplement: S3 Table — (DOCX) [file pone.0141881.s003.docx]

| **S3 Table. Complete multiple regression coefficients from main model** | | | | | | | | | | | | | |
| --- | --- | --- | --- | --- | --- | --- | --- | --- | --- | --- | --- | --- | --- |
| Model | | Unstandardized Coefficients | | Standardized Coefficients | t | Sig. | 95.0% Confidence Interval for B | | Correlations | | | Collinearity Statistics | |
|  |  | B | Std. Error | Beta |  |  | Lower Bound | Upper Bound | Zero-order | Partial | Part | Tolerance | VIF |
| 1 | (Constant) | 7.274 | .055 |  | 132.860 | .000 | 7.166 | 7.381 |  |  |  |  |  |
|  | Openness | .147 | .055 | .133 | 2.684 | .008 | .039 | .255 | .133 | .133 | .133 | 1.000 | 1.000 |
| 2 | (Constant) | 7.273 | .055 |  | 132.735 | .000 | 7.166 | 7.381 |  |  |  |  |  |
|  | Openness | .146 | .055 | .132 | 2.669 | .008 | .039 | .254 | .133 | .132 | .132 | 1.000 | 1.000 |
|  | PRI | -.031 | .054 | -.028 | -.568 | .570 | -.137 | .076 | -.031 | -.028 | -.028 | 1.000 | 1.000 |
| 3 | (Constant) | 7.269 | .054 |  | 134.470 | .000 | 7.162 | 7.375 |  |  |  |  |  |
|  | Openness | .117 | .055 | .106 | 2.143 | .033 | .010 | .225 | .133 | .107 | .105 | .976 | 1.024 |
|  | PRI | .014 | .055 | .013 | .260 | .795 | -.095 | .124 | -.031 | .013 | .013 | .921 | 1.086 |
|  | Mood | -.076 | .059 | -.067 | -1.302 | .194 | -.192 | .039 | -.106 | -.065 | -.064 | .891 | 1.122 |
|  | MW | -.176 | .056 | -.159 | -3.114 | .002 | -.287 | -.065 | -.189 | -.154 | -.152 | .916 | 1.092 |
| 4 | (Constant) | 7.263 | .053 |  | 137.203 | .000 | 7.159 | 7.367 |  |  |  |  |  |
|  | Openness | .067 | .055 | .061 | 1.226 | .221 | -.041 | .175 | .133 | .062 | .059 | .930 | 1.075 |
|  | PRI | .003 | .055 | .003 | .054 | .957 | -.106 | .112 | -.031 | .003 | .003 | .885 | 1.129 |
|  | Mood | -.121 | .060 | -.107 | -2.001 | .046 | -.240 | -.002 | -.106 | -.100 | -.096 | .803 | 1.246 |
|  | MW | -.155 | .058 | -.140 | -2.683 | .008 | -.269 | -.041 | -.189 | -.134 | -.128 | .839 | 1.192 |
|  | RC | .015 | .054 | .014 | .284 | .777 | -.090 | .121 | .022 | .014 | .014 | .919 | 1.088 |
|  | NLE | -.007 | .058 | -.007 | -.127 | .899 | -.122 | .107 | -.037 | -.006 | -.006 | .847 | 1.181 |
|  | SES | .201 | .056 | .183 | 3.617 | .000 | .092 | .311 | .192 | .179 | .173 | .894 | 1.118 |
|  | CFS | .191 | .057 | .173 | 3.361 | .001 | .079 | .302 | .105 | .167 | .161 | .862 | 1.161 |
| 5 | (Constant) | 7.262 | .053 |  | 137.056 | .000 | 7.158 | 7.366 |  |  |  |  |  |
|  | Openness | .064 | .055 | .058 | 1.164 | .245 | -.044 | .173 | .133 | .059 | .056 | .920 | 1.087 |
|  | PRI | .001 | .056 | .001 | .011 | .991 | -.109 | .110 | -.031 | .001 | .001 | .879 | 1.137 |
|  | Mood | -.110 | .064 | -.097 | -1.699 | .090 | -.236 | .017 | -.106 | -.085 | -.081 | .708 | 1.413 |
|  | MW | -.150 | .059 | -.135 | -2.553 | .011 | -.265 | -.034 | -.189 | -.128 | -.122 | .815 | 1.228 |
|  | RC | .014 | .054 | .013 | .267 | .789 | -.091 | .120 | .022 | .013 | .013 | .918 | 1.089 |
|  | NLE | -.005 | .059 | -.005 | -.094 | .925 | -.121 | .110 | -.037 | -.005 | -.004 | .844 | 1.185 |
|  | SES | .197 | .056 | .179 | 3.501 | .001 | .086 | .308 | .192 | .174 | .168 | .876 | 1.142 |
|  | CFS | .189 | .057 | .172 | 3.331 | .001 | .078 | .301 | .105 | .166 | .159 | .860 | 1.163 |
|  | Maternal characteristics | .032 | .062 | .028 | .519 | .604 | -.090 | .154 | .135 | .026 | .025 | .773 | 1.294 |
| 6 | (Constant) | 7.247 | .054 |  | 134.473 | .000 | 7.141 | 7.353 |  |  |  |  |  |
|  | Openness | .061 | .055 | .055 | 1.102 | .271 | -.048 | .169 | .133 | .056 | .052 | .906 | 1.104 |
|  | PRI | -.007 | .056 | -.006 | -.126 | .900 | -.116 | .102 | -.031 | -.006 | -.006 | .860 | 1.163 |
|  | Mood | -.097 | .064 | -.086 | -1.517 | .130 | -.223 | .029 | -.106 | -.077 | -.072 | .702 | 1.424 |
|  | MW | -.145 | .058 | -.131 | -2.492 | .013 | -.259 | -.031 | -.189 | -.125 | -.118 | .813 | 1.230 |
|  | RC | .006 | .053 | .005 | .109 | .913 | -.099 | .110 | .022 | .006 | .005 | .915 | 1.093 |
|  | NLE | .003 | .058 | .002 | .045 | .964 | -.112 | .117 | -.037 | .002 | .002 | .839 | 1.192 |
|  | SES | .207 | .056 | .188 | 3.713 | .000 | .097 | .317 | .192 | .185 | .176 | .872 | 1.147 |
|  | CFS | .187 | .056 | .170 | 3.327 | .001 | .077 | .298 | .105 | .166 | .157 | .857 | 1.168 |
|  | Maternal characteristics | .032 | .062 | .028 | .515 | .607 | -.089 | .153 | .135 | .026 | .024 | .764 | 1.309 |
|  | NLE*mat char | .087 | .057 | .077 | 1.529 | .127 | -.025 | .198 | .058 | .077 | .072 | .888 | 1.126 |
|  | SES* mat char | .135 | .055 | .119 | 2.447 | .015 | .027 | .244 | .087 | .123 | .116 | .941 | 1.063 |
|  | RC* mat char | -.136 | .054 | -.124 | -2.534 | .012 | -.242 | -.031 | -.104 | -.127 | -.120 | .933 | 1.072 |
|  | CFS * mat char | -.076 | .056 | -.070 | -1.354 | .177 | -.187 | .034 | -.057 | -.068 | -.064 | .830 | 1.205 |
| Note: PRI = Pregnancy Risk Index, NLE = Negative Life Events, SES = Socio-economic Status, CFS = Chronic Family Stress, RC = Relational Conflict, MW = Material Worry. | | | | | | | | | | | | | |
